# Brownian Ratchet Mechanism for Faithful Segregation of Low-Copy-Number Plasmids

**DOI:** 10.1016/j.bpj.2017.02.039

**Published:** 2017-04-11

**Authors:** Longhua Hu, Anthony G. Vecchiarelli, Kiyoshi Mizuuchi, Keir C. Neuman, Jian Liu

**Affiliations:** 1 Biochemistry and Biophysics Center, National Heart, Lung and Blood Institute, National Institutes of Health, Bethesda, Maryland; 2 Laboratory of Molecular Biology, National Institute of Diabetes and Digestive and Kidney Diseases, National Institutes of Health, Bethesda, Maryland; 3 Department of Molecular, Cellular, and Developmental Biology (MCDB), University of Michigan, Ann Arbor, Michigan

## Abstract

Bacterial plasmids are extrachromosomal DNA that provides selective advantages for bacterial survival. Plasmid partitioning can be remarkably robust. For high-copy-number plasmids, diffusion ensures that both daughter cells inherit plasmids after cell division. In contrast, most low-copy-number plasmids need to be actively partitioned by a conserved tripartite ParA-type system. ParA is an ATPase that binds to chromosomal DNA; ParB is the stimulator of the ParA ATPase and specifically binds to the plasmid at a centromere-like site, *parS*. ParB stimulation of the ParA ATPase releases ParA from the bacterial chromosome, after which it takes a long time to reset its DNA-binding affinity. We previously demonstrated in vitro that the ParA system can exploit this biochemical asymmetry for directed cargo transport. Multiple ParA-ParB bonds can bridge a *parS-*coated cargo to a DNA carpet, and they can work collectively as a Brownian ratchet that directs persistent cargo movement with a ParA-depletion zone trailing behind. By extending this model, we suggest that a similar Brownian ratchet mechanism recapitulates the full range of actively segregated plasmid motilities observed in vivo. We demonstrate that plasmid motility is tuned as the replenishment rate of the ParA-depletion zone progressively increases relative to the cargo speed, evolving from diffusion to pole-to-pole oscillation, local excursions, and, finally, immobility. When the plasmid replicates, the daughters largely display motilities similar to that of their mother, except that when the single-focus progenitor is locally excursive, the daughter foci undergo directed segregation. We show that directed segregation maximizes the fidelity of plasmid partition. Given that local excursion and directed segregation are the most commonly observed modes of plasmid motility in vivo, we suggest that the operation of the ParA-type partition system has been shaped by evolution for high fidelity of plasmid segregation.

## Introduction

Bacterial plasmids are extrachromosomal DNA that undergoes horizontal gene transfer within a population of microbes (1). The maintenance of bacterial plasmids during the cell cycle can endow the cells with selective advantages (1). For high-copy plasmids, random diffusion might be sufficient to ensure that both daughter cells inherit the plasmid after division (2). However, for low-copy plasmids, such as the P1 or F plasmids, active segregation machinery is needed for stable plasmid maintenance within a bacterial cell population (3, 4). These plasmids encode their own partitioning (or *par*) genes to assure that the plasmid copies are segregated, transported, and positioned along the nucleoid—a rod-like structure consisting primarily of condensed chromosomal DNA (3). The most common partition machinery is a tripartite system consisting of two proteins, ParA and ParB, and a *cis*-acting centromere-like site on the plasmid, *parS* (3, 4). This partition machinery works so well that the segregation error rate is extremely low (∼10^−3^–10^−7^ per generation) (5). ParA is an adenosine triphosphatase (ATPase) that, in an ATP-bound dimeric state, non-specifically binds DNA in vitro, and hence to the nucleoid in vivo. ParB is a dimeric protein that specifically binds to *parS*. Additional ParB molecules have been observed to bind sequence non-specifically around *parS*. When multiple copies of the plasmid exist inside a cell, ParB-bound plasmids form aggregated plasmid clusters held together by a large number of ParB molecules. We call these large ParB-plasmid complexes partition complexes (PCs). Plasmid-bound ParB can interact with nucleoid-bound ParA, which stimulates its ATPase activity and releases ParA from nucleoid (6). Whereas these biochemical reactions of the ParA-type system are essential for plasmids partitioning along the long-axis of the nucleoid, it is not understood how the chemical energy provided by ATP hydrolysis is harnessed to ensure plasmid segregation fidelity. ParA-mediated plasmid partitioning is also a prototype for the study of bacterial chromosome segregation that employs a similar tripartite positioning system and presumably a similar mechanism. Understanding the partitioning mechanism could thus provide insight into how evolution shapes more complex systems required for sub-cellular organization and positioning.

Salient features of ParA-mediated plasmid movements expose some clues of the inner workings. The key observations are as follows. PCs of some low-copy-number plasmids have been observed to undergo pole-to-pole oscillations over the nucleoid region of the cell (7, 8). However, very different PC motility patterns have been observed for a variety of plasmid systems (9, 10, 11, 12, 13, 14, 15). Many PCs of low-copy-number plasmids are mobile and frequently switch the direction of movement over the nucleoid. In most cases, rather than making large-scale oscillations between the nucleoid poles, a PC’s overall excursion from its average position appears to be limited to a small fraction of the nucleoid length (15). When only one PC is present, its average position over time remains around mid-nucleoid, which is also the mid-cell position when one nucleoid is present (15). After PC splitting, the sister PCs have been observed to persistently move apart along the nucleoid and eventually position around the cell quarter positions (8, 9, 10, 11, 12, 13, 14, 15). When more than two PCs share the same nucleoid, a new regular inter-spacing is established among them. It appears that this phenomenon is conserved across different ParA-type systems (7, 8, 15). These observations raise several questions: Why do PCs display oscillatory movement only in a subset of cells (11)? Do these diverse motility patterns reflect distinct underlying mechanisms that fulfill different functional needs? Or do they conform to, but reflect variations of, the operational principle of the partition machinery?

ParA proteins can polymerize into filament bundles under certain in vitro conditions (16). It has been proposed that a ParA filament-based growth/shrinkage mechanism could drive the segregation and movement of plasmids, similar to those mediated by cytoskeletal filaments (actin or microtubule) in eukaryotic cells (17, 18, 19). However, both recent in vitro reconstitution experiments and in vivo super-resolution microscopy have provided strong evidence against a filament-based plasmid movement mechanism for ParA/B systems (10, 20, 21). In addition, we have shown that the ParA-type system could work as a Brownian ratchet that rectifies the inherent asymmetry in its biochemical reactions (21, 22). Specifically, cargo-bound ParB stimulates the ATPase activity of DNA-bound ParA, which triggers the dissociation of ParA from the DNA substrate surface. The slow rate of dissociated ParA resetting its DNA-binding capability generates a ParA-depleted zone behind the moving cargo. The resulting asymmetric ParA distribution perpetuates the forward movement of the cargo. Meanwhile, transient tethering arising from the ParA-ParB contacts collectively drives forward movement of the cargo and quenches diffusive motion in orthogonal directions (22). This mechanism recapitulates in vitro reconstitution experiments in which ParB-laden microbeads displayed directed and persistent movement on a ParA-covered DNA carpet (21).

Here, we elaborate this model and find that the Brownian ratchet mechanism can explain the wide range of PC motility modes observed in vivo, including diffusive, stationary, and directed movements. For directed movement, two distinct patterns emerge, depending on the balance between the ParA-directed PC translocation speed and the refilling kinetics of the accompanying ParA-depletion zone. When the PC moves faster relative to the replenishment of the ParA-depletion zone, it undergoes pole-to-pole oscillation. This is because the nucleoid poles prevent the PC from moving farther, which allows ParA refilling to catch up and reverse the direction of PC movement. When the directed PC movement is comparable with the ParA refilling kinetics, the ParA-depletion zone is significantly diminished. In this scenario, a single PC undergoes local excursions, whereas after PC splitting, the daughter PCs undergo directed segregation along the nucleoid long axis. With random initial positioning of a single parental PC, the model shows that partition fidelity is optimal when the separation distance arising from this directed segregation is around half of the nucleoid length, positioning the sister PCs at roughly the quarter positions, as observed in most in vivo experiments (8, 9, 10, 11, 12, 13, 14, 15). We therefore suggest that plasmid pole-to-pole oscillations are rare events that reflect a deviation from this optimal control. And the observed differences in plasmid motilities reflect fluctuations in specific parameters rather than fundamentally different mechanisms of segregation plasmid motility.

## Materials and Methods

### Theoretical model

**Figure 1:**
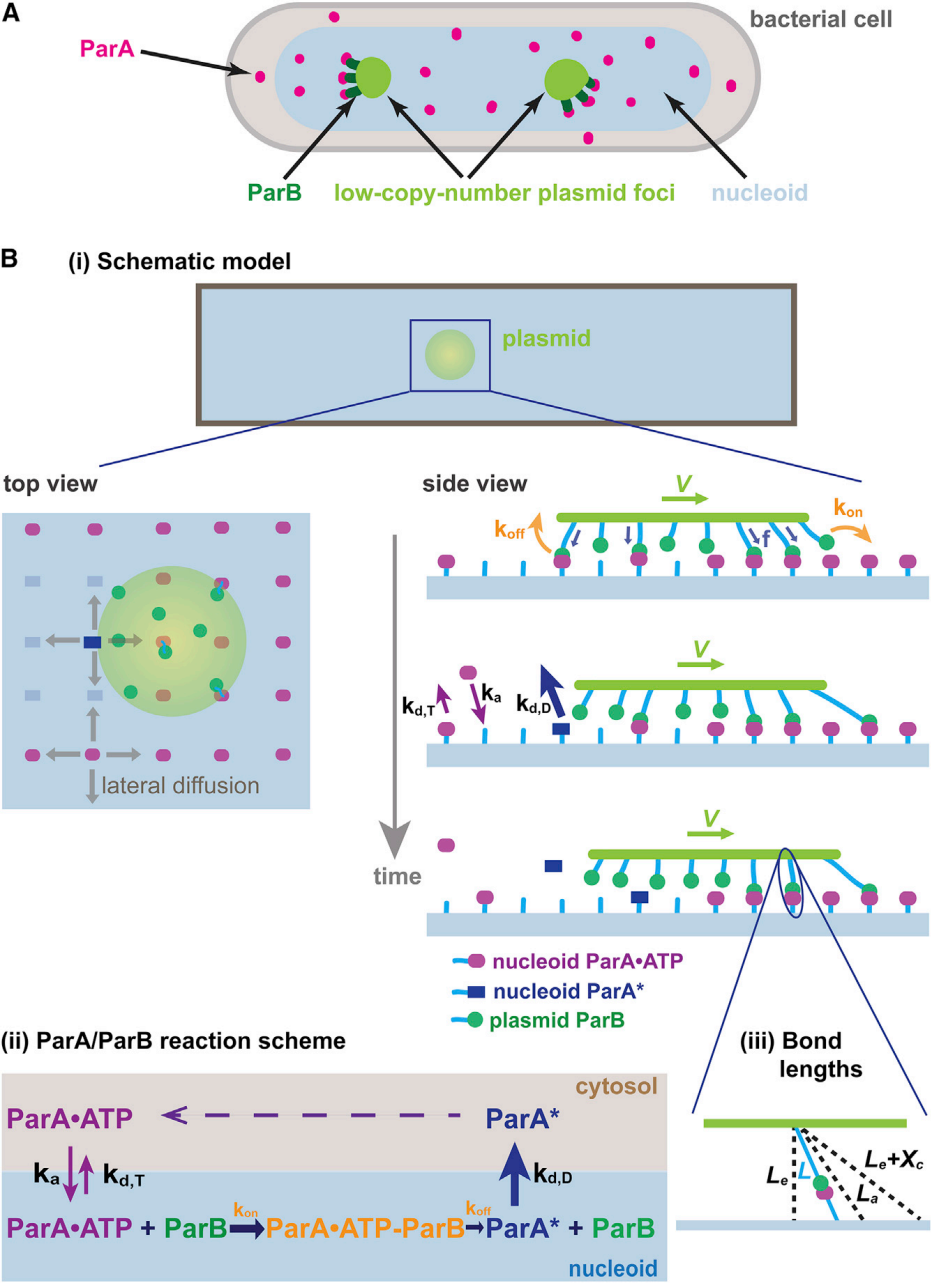
Schematic model of plasmid partitioning. (*A*) Cartoon of plasmid segregation in a bacterial cell shows the key components of ParA-mediated plasmid segregation. (*B*) Model setup: (*i*) schematic of model geometry; (*ii*) simplified ParA/ParB biochemical reaction scheme; (*iii*) definitions of lengths associated with ParA/ParB interactions.

We first extend the geometric setup and the biochemical reactions of our previous model to specify PC partitioning in vivo (as outlined in Fig. 1 *A*). As we will show, the key differences in the new setup underlie the greater diversity of motility patterns observed in vivo compared to those in vitro.

Geometrically, the model considers the simplest scheme by representing the PC as a circular disc (Fig. 1 *Bi*). Due to the limitation of the spatial resolution in current experiments, in vivo trajectories of PCs typically reflect the projection of 3-D images onto a 2-D plane of the nucleoid. Therefore, we consider a simple 2-D model for practical purposes and represent the nucleoid as a flat rectangle (Fig. 1 *Bi*). Lengthwise, the model imposes a hard-wall boundary condition. As a simplification, we also treat the lateral boundaries as hard walls. We note that the model is insensitive to the precise dimensions of the nucleoid; as long as the width is shorter than the length, simulation domains of different widths do not critically impact the model results (Fig. S1). For simplicity, we will refer to the circular disc and the rectangular substrate in the model as a PC and a nucleoid, respectively. In this context, ParA·ATP dimers bind all over the nucleoid surface, whereas ParB dimers coalesce on the plasmid (Fig. 1 *Bi*).

From a biochemical perspective, we improve on the previous model by incorporating the exchange between the cytosolic and the nucleoid-bound ParA·ATP, as well as the diffusion of ParA·ATP on the nucleoid surface (Fig. 1 *Bii*) (22). We treat the cytosolic pool as an implicit reservoir that dictates the basal rates of ParA·ATP binding to the nucleoid, in which the binding event only occurs at locations on the nucleoid that are unoccupied by ParA. Along the nucleoid, ParA·ATP that does not interact with ParB can transiently release from an occupied site and bind an adjacent vacant site on the nucleoid via lateral diffusion (23). Upon binding to PC-bound ParB, ParA can no longer diffuse. As defined in our previous model (22), deformation of the ParA-ParB bond, similar to stretching or compressing of a spring, generates restoring forces on the PC (Fig. 1 *Bi*). Note that the entire tether bridging the nucleoid and the plasmid, i.e., the nucleoid DNA-ParA·ATP-ParB-plasmid linkage, shares the associated deformation. Only for brevity, we define the ParA-ParB bond as representing this series of connected linkages. The vector sum of many ParA·ATP-ParB bonds across the PC collectively generates a net force that displaces the PC, which resets the bond configurations. When random events (e.g., PC diffusion and stochastic ParA·ATP-ParB bond dynamics) break symmetry, the PC moves forward with the ParA·ATP-ParB bonds broken at its back. We define the resulting disengaged ParA to be in a distinctive state, ParA^∗^. As we currently do not yet know the detailed biochemical mechanism of ParB-stimulated ATPase activity of ParA in the presence of DNA, the model does not specify whether ParA^∗^ corresponds to an ATP-bound or ADP-bound state. The model nevertheless describes two key aspects of disengaged ParA. First, ParA^∗^ dissociates from the nucleoid faster than the basal turnover rate of ParA·ATP (Fig. 1 *Bii*), which reflects the known effect of ParB-mediated stimulation on ParA release from the nucleoid (23). Second, once ParA^∗^ dissociates into the cytosol, it slowly converts back to the ATP-bound state that is competent for DNA-binding (23). This time delay results in a ParA-depletion zone trailing behind the moving PC, which subsequently can be refilled by cytosolic ParA·ATP and lateral diffusion of nucleoid-associated ParA·ATP. As the PC moves forward, ParB on the leading edge of the PC continues to establish new bonds with ParA·ATP on unexplored regions of the nucleoid, where the ParA·ATP concentration is higher. PC movement therefore maintains the asymmetric biochemical environment that in turn supports further forward movement. Conceptually, our model is similar to burnt-bridge Brownian ratchet models (24, 25). The difference is that previous models concern one-dimensional problems (24, 25), which by definition do not consider lateral excursions orthogonal to the direction of motion. In contrast, our model addresses the more realistic two-dimensional case. Mechanical actions of the multiple bonds in our case not only facilitate forward movement of the cargo, but also collectively provide tethering that quenches lateral diffusion, from which directed and persistent movement emerges (22).

To quantitatively elucidate the proposed mechanism, we numerically compute our model with geometric and kinetic parameters that mimic in vivo conditions. Instead of focusing on a specific model system, we chose a generic set of model parameters that reflects common features shared among different ParA-type plasmid partition systems. Whereas the quantitative characteristics vary considerably among these systems, we note that the qualitative essence of our model results presented below remains robust. The default dimensions of the nucleoid in the simulation are 2 *μ*m in length and 1 *μ*m in width. The nucleoid-bound ParA·ATP dimers are initially in chemical equilibrium with their cytosolic counterparts and are randomly distributed on the nucleoid lattice with 5 nm spacing. ParB dimers were permanently distributed with a uniform density of 0.05 ParB dimer/nm^2^ over the PC, which has a radius of 100 nm (26, 27). The high density of plasmid-bound ParB reflects the known propensity of ParB to spread and cluster around the *parS* site on the plasmid (28, 29, 30). We model each ParA·ATP-ParB bond as an elastic spring. This spring in effect is the nucleoid DNA-ParA·ATP-ParB-plasmid linkage, whose spring constant is mainly determined by the softest component—the nucleoid (see Supporting Material for details). The vertical distance between the nucleoid and the PC was fixed at the equilibrium bond length of ParA·ATP-ParB (*L*_e_) throughout the simulation (Fig. 1 *Biii*). The subsequent stochastic reactions between plasmid-bound ParB and nucleoid-bound and cytosolic ParA are simulated with the kinetic Monte Carlo scheme in accordance with the reaction scheme illustrated in Fig. 1 *Bii*. At each simulation time step, each ParB dimer can interact with available ParA·ATP within a distance *L*_a_, and bind only one ParA at a time. The probability of binding is proportional to $\text{exp}\left(-\left(\left(1/2\right)k_{\text{s}}{\left(L-L_{\text{e}}\right)}^{2}/k_{\text{B}}T\right)\right)$ for $L_{\text{a}}\geq L\geq L_{\text{e}}$; otherwise, it is zero. Here, *k*_s_ is the spring constant of the bond. *L* denotes the separation between ParB and ParA·ATP (Fig. 1 *Biii*). If this bond forms, *L* is the instantaneous bond length, and $\left(1/2\right)k_{\text{s}}{\left(L-L_{\text{e}}\right)}^{2}$ represents the associated elastic energy penalty. Importantly, given the model parameters (see Table S1), the maximum of this energy penalty is less than the thermal energy, *k*_B_T. Consequently, thermal energy is sufficient to pre-stretch the newly formed bond, which in turn provides an elastic force. In the simulation, we vector-sum the elastic forces from all the ParA·ATP-ParB bonds over the plasmid. This net force, in addition to diffusion, drives PC motion for one time step. The model ignores forces in the *z*-direction, which are assumed to be balanced due to the confined space between the cell membrane and the nucleoid surface in vivo. In the next time step, the bond lengths of the ParA·ATP-ParB complexes are updated by this motion, from which the dissociation rates of the existing ParA·ATP-ParB complexes are calculated according to the following force dependence: when the bond extension (*L* − *L*_e_) > *X*_C_ (Fig. 1 *Biii*), where *L* is the instantaneous bond length, the bond breaks instantaneously; otherwise, the dissociation rate is determined from $k_{\text{off}}\;\left(f\right)=k_{\text{off}}^{0}e^{\left(f/fc\right)},$ where $k_{\text{off}}^{0}$ is the intrinsic dissociation rate of the bond; *f* is the elastic force stemming from the bond stretching, *f* = *k*_s_(*L* − *L*_e_); and *f*_c_ is the characteristic bond-dissociation force. This generic force-dependent dissociation rate was used for the simulations; however, the model results are relatively insensitive to the specific form of the force dependence of the dissociation rate (22). This dissociation reaction is next implemented in the stochastic simulation. Meanwhile, PC movement from the previous time step permits unbound ParBs to explore new territory and form bonds with available ParA·ATPs. In addition, vacancies on the nucleoid can be re-filled by ParA·ATP rebinding from the cytosol or diffusing from adjacent sites on the nucleoid. These ParA·ATPs can establish new bonds with ParB if the PC is nearby. We then update the net force from all the ParA·ATP-ParB bonds, including changes in existing bonds and newly formed bonds. The movement of the cargo is then calculated as in the previous time step. We repeat these steps throughout the simulation over time.

**Figure 2:**
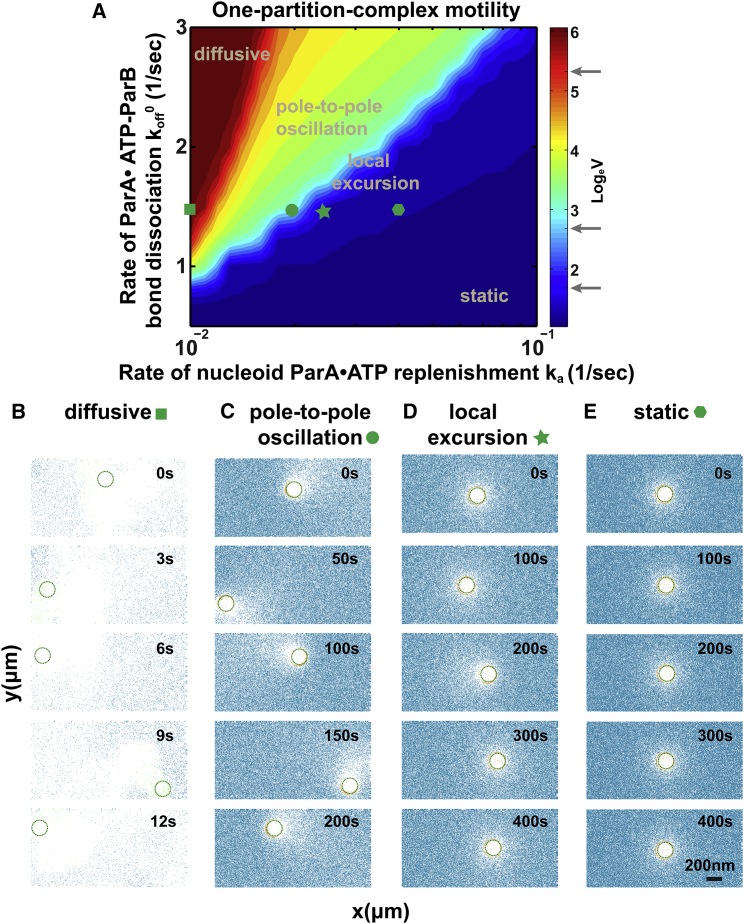
Distinct motility patterns of a single PC. (*A*) Phase diagram of single-PC movement. The color map reflects the log scale of the average speed (*V*, nm/s) of the simulated PC movement over 10 min; it is used as a metric to represent the gradual transition between different motility patterns. We simulate PC motility while varying the ParA·ATP-ParB bond dissociation rate, *k*_off_^0^, and the ParA refilling rate, *k*_a_, while keeping the remaining parameters at their respective nominal values (see Table S1). (*B*–*E*) Representative dynamic trajectories of a single PC undergoing diffusion (*B*), pole-to-pole oscillation movement (*C*), local excursion around the average position (*D*), and being static (*E*). For (*B*)–(*E*), the corresponding sets of parameter values for each representative trajectory are labeled in (*A*); and the corresponding ParA spatial profile is overlaid with the PC position in each snapshot. ParA·ATP is shown in blue-green, ParA^∗^ in green, ParA·ATP-ParB in orange, and the vacant site in white. We note that motility patterns of plasmids are multi-faceted and in general are difficult to describe with a single parameter. From our simulations, we noticed that the movement speed correlated with the distinct motility patterns we describe. The average speed of diffusive motion is in general ill defined. Nonetheless, for the purpose of comparing among modes in the phase diagram, we define the average speed in diffusive motions as the traveled distance divided by a fixed traveling time of 10 min. We used average movement speed as a continuous metric to infer discrete movement types, which are delineated by the gray arrows in the color bar in (*A*) (see Fig. S4 for details).

**Figure 3:**
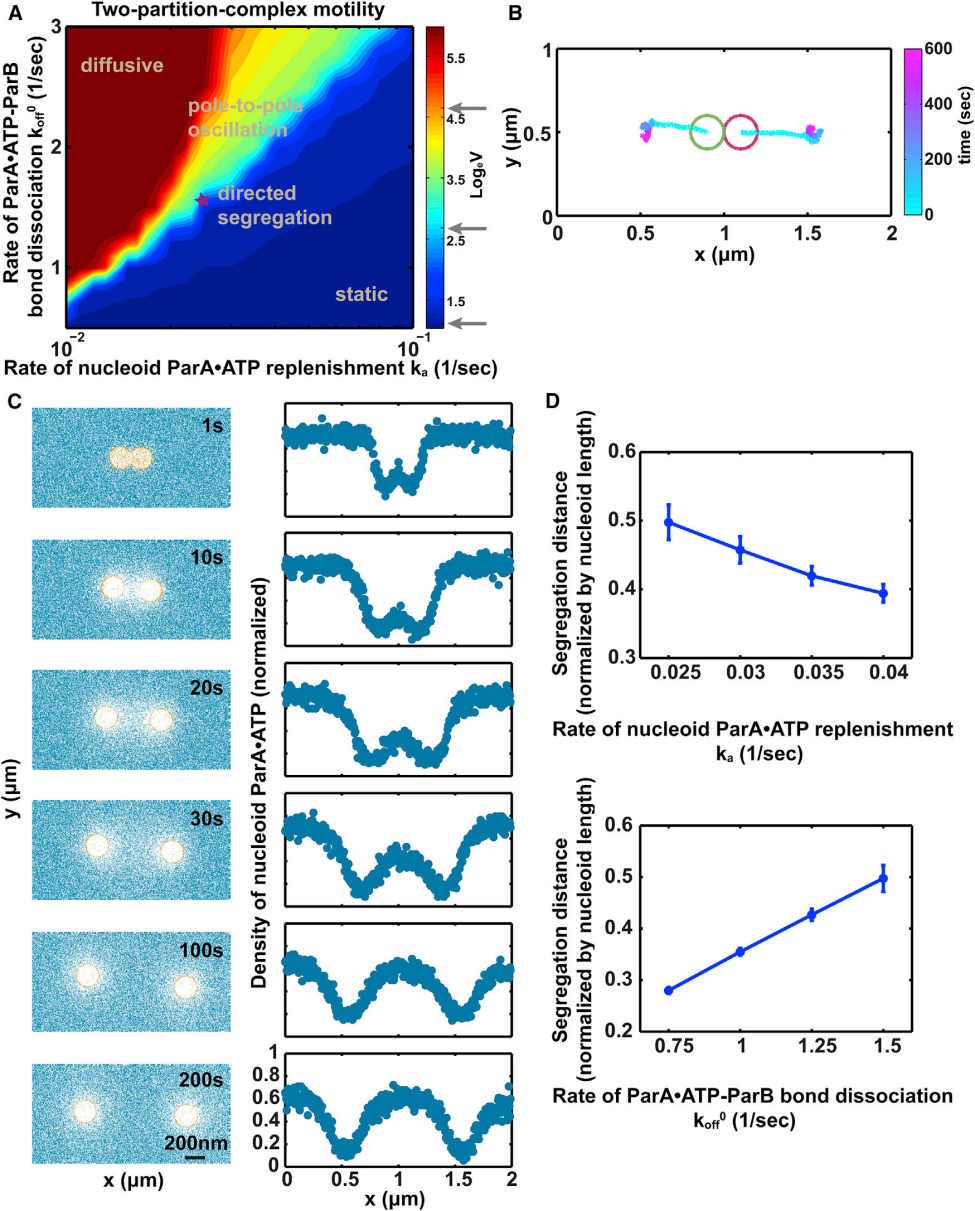
Directed segregation of two PCs. (*A*) Phase diagram of two-PC movements. The color map reflects the log scale of the average speed (*V*, nm/s) of the simulated PC movement over 10 min; it is used as a surrogate to represent the gradual transition between different motility patterns. We simulate the PC motility starting with both foci at the mid-position along the nucleoid. We vary the ParA·ATP-ParB bond dissociation rate, *k*_off_^0^, and the ParA refilling rate *k*_a_, while keeping all the other parameters at their respective nominal values (see Table S1). (*B*) A representative dynamic trajectory of directed-segregation motion illustrating the positions along (*x* axis) and across (*y* axis) the nucleoid as a function of time (represented by the varying color of the trajectory). (*C*) PCs move toward regions of higher ParA concentration until the spatial distributions surrounding the PCs are symmetric. On the substrate surface, ParA·ATP is shown in blue-green, ParA^∗^ in green, and ParA·ATP-ParB in orange. For (*B*) and (*C*), the parameter set corresponds to those labeled with a star in (*A*). (*D*) Dependence of dual PC separation distance on the relative rates of ParA refilling and ParA·ATP-ParB bond dissociation. The rate of ParA·ATP-ParB bond dissociation and ParA refilling are fixed in the corresponding plots as the nominal values (see Table S1). Like the phase diagram in Fig. 2*A*, we used average movement speed as a continuous proxy to infer discrete movement types, which are delineated by the gray arrows in the color bar in (*A*) (see Fig. S4 for details).

The model parameters are estimated from existing experimental measurements wherever possible (see Table S1). We follow the same simulation scheme as in our previous work (22), where the Brownian dynamics time step is set to 10^−5^ s, which is short enough to account for the fastest reaction/diffusion process in the system. Below, we present typical model results, of which the essence is preserved over a broad range of model parameter space, as demonstrated by our phase diagram studies (Figs. 2, 3, and S1–S3).

## Results

### Single-PC motility modes

To discern the underlying mechanism of PC movement, we start with the simplest case, in which there is a single PC on the nucleoid. Our simulation begins with the following initial conditions: 1) the PC is positioned at the center of the nucleoid; 2) ParA·ATP dimers are randomly distributed on the nucleoid and are in equilibrium with the cytosolic counterpart; and 3) the plasmid-bound ParB dimers have not yet established any bonds with ParA·ATP. We obtain the subsequent dynamics of the system by stochastic simulations of the model.

Our previous in vitro work suggests that after symmetry breaking, rather than being diffusion-limited, the directed and persistent cargo movement speed is controlled by ParA-ParB bond dissociation (22). To investigate how the Brownian ratchet mechanism plays out in vivo, we focus on its key distinction from the in vitro conditions. The crucial difference is the refilling of ParA in vivo, which is due to the confined space inside a bacterial cell and the limited amount of total protein. We thus set out to explore the effects of ParA refilling on PC motility. Fig. 2 *A* presents the calculated phase diagram describing the dependence of PC movements on the ParA-ParB bond dissociation rate and the rate of ParA refilling from the cytosol onto the nucleoid. Notably, the model recapitulates the full range of observed motility modes of the PC: diffusion, pole-to-pole oscillation, local excursion, and being stationary (Fig. 2, *A–E*) (7, 8, 9, 10, 11, 12, 13, 14, 15). The transitions between the neighboring motility phases are continuous rather than abrupt. The snapshots in Fig. 2, *B–E*, illustrate representative PC movement trajectories and the corresponding ParA spatial profile for each phase. When ParA-ParB bond dissociation is fast and ParA rebinding is slow, the overall steady-state condition dictates that the density of nucleoid-bound ParA is low (Fig. 2 *B*). Consequently, the PC displays random diffusion (Fig. 2, *A* and *B*). This reflects the insufficient number of ParA-ParB tethers, which are needed to quench cargo diffusion, consistent with the notion put forward by our previous results (22). This prediction is also in line with in vivo observations, e.g., the highly mobile movement of the plasmid RK2 without its Par system in *Escherichia coli* (31). Conversely, with a very low ParA-ParB bond dissociation rate and a fast ParA re-binding to the nucleoid, the PC remains essentially stuck. This is because the nucleoid-bound ParA concentration remains high and symmetric around the plasmid; consequently, the PC is not able to break symmetry that sets the movement in the first place (Fig. 2 *E*).

Between the stationary and diffusive regimes, a single PC could display pole-to-pole oscillations or local excursions, depending on the balance between the ParA refilling rate and the ParA-ParB bond dissociation rate (Fig. 2 *A*). When the bond dissociation rate is high, the PC movement is faster than the pace of ParA refilling; the PC undergoes directed and persistent movement after symmetry breaking, leaving a significant ParA-depletion zone in its wake (Fig. 2 *C*). Only when the PC approaches a nucleoid boundary near the pole, beyond which it cannot proceed, can ParA refilling catch up and cause the PC to move toward the opposite pole. In these oscillatory events, it appears that the PC “chases” the retreating ParA concentration gradient, recapitulating the observed ParA profiles during PC oscillation in vivo (e.g., in (8)).

It should also be noted that the PC does not have to reach the nucleoid pole to reverse direction. As the system approaches the stationary regime from the pole-to-pole regime in the phase diagram, an intermediate behavior emerges: The PC reverses direction before reaching the pole (Fig. 2 *D*). Under these conditions, it appears that the PC displays small excursions around its average position, mimicking some in vivo observations (15, 32). The trailing ParA-depletion zone facilitates PC forward movement, whereas ParA refilling smoothes the depletion zone, re-establishes symmetry, and traps the PC. In this intermediate regime, the ParA-depletion zone is significantly diminished, because the rate of PC movement roughly balances out that of the ParA refilling. Consequently, effects of stochastic fluctuations become more prominent. Noise thus renders the PC motion more stochastic, driving motion of the PC at one instant and trapping it at another. The resulting local excursions are similar to the saltatory movement demonstrated by our previous in vitro simulations and experiments (22) (Fig. 2 *D*).

### Directed segregation of two plasmids

Following the above results, we next ask: How does the same Brownian ratchet mechanism play out with multiple PCs sharing the same nucleoid? Does it result in PC segregation? We begin to address these questions by studying the two-PC case pertaining to plasmid replicas in vivo. After plasmid replication, the sister plasmids transiently stay together, possibly due to *trans* ParB-ParB interactions (33). This cohesion is unstable and appears to be readily destroyed by random fluctuations (15). Since the model does not include ParB-ParB interactions, we make the simplifying assumption that the sister plasmid foci have already split into independent entities and are poised side by side along the long axis at the center of the nucleoid. We further assume that there is volume exclusion between the foci.

Fig. 3 *A* shows that whereas the two PCs display similar motility to that of their single-PC progenitor, a new phenomenon emerged when the parental PC was undergoing local excursions (Figs. 2 *A* and 3 *A*). We term this new behavior “directed segregation”: the sister PCs initially move apart persistently toward their respective poles; after a critical separation distance, they become relatively stationary, with small excursions around more or less symmetric positions with respect to mid-nucleoid (Fig. 3 *B*). This recapitulates the partitioning of low-copy-number plasmids after PC splitting, as observed in vivo (8, 9, 10, 11, 12, 13, 14, 15). This dynamic pattern arises under the same conditions for which the single PC exhibits local excursions (Figs. 2 *A* and 3 *A*), wherein the replenishment rate of the ParA-depletion zone is comparable to the plasmid movement speed. What makes the difference in the two-PC case is the initial condition. As shown in our previous work (22), the symmetry-breaking event entails that the ParB-laden cargo depletes enough of the ParA·ATPs that the remaining ParA-ParB bond tetherings become insufficient to quench the stochastic fluctuations of the cargo. At this stage, once the cargo moves in one direction, it will leave behind the ParA-depletion zone while chasing the higher ParA·ATP concentration at its front, thus breaking symmetry. With two side-by-side PCs, volume-exclusion effects are exerted on both foci, which serves to break the symmetry of the system and drive directed motion. This symmetry breaking drives the two foci apart until the trailing ParA-depletion zones are refilled. Concomitantly, symmetric distributions of ParA concentration surrounding each PC are re-established (Fig. 3 *C*), in line with observations in earlier works (11, 34). The individual PCs subsequently behave just as in the single-PC case, undergoing limited excursions.

To gain further insight into this interesting motility pattern, we carried out more quantitative analysis of the motility. As the analytic solutions from our previous work show (22), after symmetry breaking, the directed speed at steady state is proportional to the bond-dissociation rate, $k_{\text{off}}^{0}$, and independent of cargo diffusion: $V∼k_{\text{off}}^{0}\left(L_{\text{a}}-L_{\text{e}}\right),$ where *L*_e_ is the equilibrium bond length and *L*_a_ is the maximal bond length for bond formation (see Fig. 1 *Biii*). In the context presented here, the factor opposing directed movement is ParA refilling onto the ParA-depletion zone. As a very rough estimate, the refilling speed is ∼${\tilde{k}}_{\text{a}}\Delta$, where Δ is the lattice spacing in the model and ${\tilde{k}}_{\text{a}}$ is the rate of effective ParA refilling, which combines the lateral ParA diffusion along the substrate and the deposition of ParA directly from the cytoplasm. The time it takes for the ParA refilling to catch up with the forward-moving PC is approximately: $2R/\left({\tilde{k}}_{\text{a}}\Delta -k_{\text{off}}^{0}\left(L_{\text{a}}-L_{\text{e}}\right)\right)$, where 2*R* is the diameter of the PC, which is roughly the linear dimension of the initial ParA-depletion zone. Therefore, if we assume that the velocity is constant throughout the separation process, the separation distance will be the separation duration times the relative speed between the two PCs. We can thus obtain a very rough estimate on the separation distance as ∼$\left(4Rk_{\text{off}}^{0}\;\left(L_{\text{a}}-L_{\text{e}}\right)/\left({\tilde{k}}_{\text{a}}\Delta -k_{\text{off}}^{0}\;\left(L_{\text{a}}-L_{\text{e}}\right)\right)\right)$. Note that this formula only applies to the scenario in which the overall refilling speed, ${\tilde{k}}_{\text{a}}\Delta$, is faster than the forward movement speed, $k_{\text{off}}^{0}\;\left(L_{\text{a}}-L_{\text{e}}\right)$. This semi-quantitative relation indicates that the faster the ParA-ParB bond dissociates, the faster the PC moves, the more the separation distance increases. Conversely, increasing the effective ParA refilling rate will speed up the catch-up and shorten the separation distance. Of course, when the forward movement speed is faster than the refilling rate, i.e., $k_{\text{off}}^{0}\;\left(L_{\text{a}}-L_{\text{e}}\right)>{\tilde{k}}_{\text{a}}\Delta$, the above formula does not hold up anymore. Nevertheless, the directed PC will perpetuate its movement until hitting the nucleoid pole and subsequently undergo pole-to-pole oscillation. Indeed, our numerical simulation supports these insights. It shows that the resulting separation distance between the two PCs hinges on the relative rate of ParA-nucleoid rebinding with respect to the PC movement speed (Fig. 3 *D*). The phase diagram illustrates that as the system approaches the stationary regime, the depletion zone is refilled faster (Fig. 3 *A*) and the separation distance between sister PCs decreases (Fig. 3 *D*). Conversely, this separation distance increases as the system moves from the stationary-phase regime toward the oscillatory-phase regime (Fig. 3 *D*).

### Fidelity of plasmid segregation

PC motility ultimately confers plasmid segregation before cell division, which is remarkably precise and robust (5). From this functional perspective, we next leverage our model to investigate how different motility modes predicted by our simulation in different regimes perform in terms of plasmid partition fidelity. We focus on the two-PC case. Since the plasmid replicates independently of cell division (35, 36, 37), and PC splitting could take place any time prior to this event (38), we gauge partition fidelity by the segregation probability. We define the segregation probability as the percentage of time that the two PCs are located on different halves of the same nucleoid. For each set of model parameters, this segregation probability is averaged from >30 dynamic trajectories of independent stochastic simulation runs. We note that we use this segregation probability as a proxy to differentiate the effects of different PC motility modes on plasmid partition fidelity, rather than as a measure that can be quantitatively compared with experiments. In this sense, higher segregation probability can be inferred to indicate more faithful plasmid partitioning.

**Figure 4:**
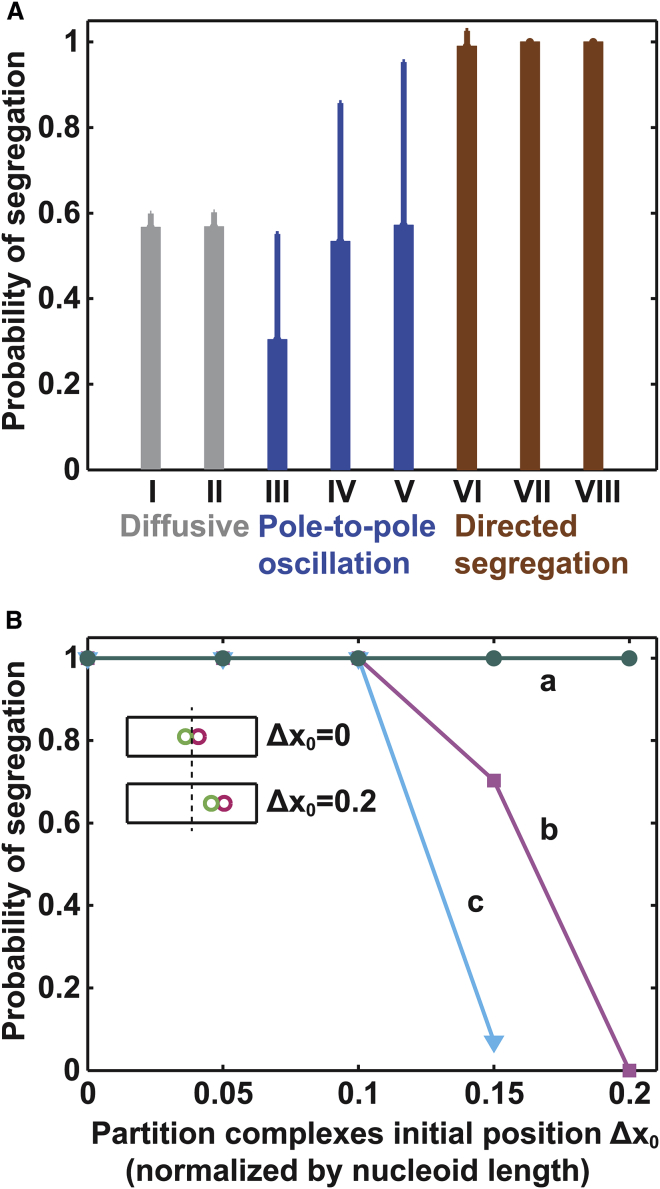
Directed segregation improves fidelity of PC segregation. (*A*) Segregation fidelity determined for different modes of PC motility. Each case corresponds to a different set of model parameters in the phase diagram of Fig. 3*A*. Specifically, the ParA·ATP-ParB bond dissociation rate, *k*_off_^0^, is fixed at 2.5/s for all cases, whereas the ParA refilling rate, *k*_a_, is varied: *k*_a_ = 0.02/s (*I*) and 0.025/s (*II*) for diffusive movements; *k*_a_ = 0.035/s (*III*), 0.04/s (*IV*), and 0.05/s (*V*) for pole-to-pole oscillation; and *k*_a_ = 0.06/s (*VI*), 0.08/s (*VII*), and 0.1/s (*VIII*) for directed segregation. For each case, we describe its statistics by the average ± SD over 36 stochastic simulation runs with the same set of parameters, where each run simulates 10 min of PC movement. (*B*) Dependence of partition fidelity on the initial positions of PCs and the separation distance in the directed-segregation mode. Here, the separation distance is *a* > *b* > *c*. For curves *a*–*c*, we calculate the partition probability using the parameter sets in the phase diagram of Fig. 3*A* with *k*_a_ = 0.028/s, 0.06/s, and 0.08/s, respectively, while keeping *k*_off_^0^ = 1.5/s. These parameter sets yield the corresponding separation distances of 0.948, 0.657, and 0.582 *μ*m, respectively. The inset of (*B*) illustrates the definition of the initial position of PCs in the model.

Fig. 4 *A* summarizes our stochastic simulation results. The random nature of diffusion understandably yields ∼50% segregation probability. The pole-to-pole oscillation mode does not notably improve the fidelity and exhibits large variability. In this case, the two PCs keep on moving in a directed and persistent manner. Compared with diffusive movement, the two PCs associate with a much more significant ParA-depletion zone behind them, and hence they could effectively interact via their “tails.” When they move toward each other in an exact end-on collision course, the PCs will repel each other and “bounce” back. Due to the everlasting stochastic fluctuations, however, what frequently happens is that the two PCs are not exactly head-on when they collide. Consequently, the two PCs may travel together in the direction of the vector sum (Fig. S5). The two associated ParA-depletion zones will now coalesce into one coherent ParA-depletion zone (Fig. S5), which perpetuates the forward movement of both PCs, keeping them together for an extended period of time. This reduces the segregation probability. As a result, the segregation probability exhibits a large uncertainty (Fig. 4 *A*). This suggests that the pole-to-pole oscillation regime may not yield faithful partitioning in a robust fashion.

In contrast, directed segregation could provide a more controllable and reliable means to ensure segregation fidelity (Fig. 4 *A*). Directed segregation of PCs typically takes approximately minutes (Fig. 3 *B*), which is much faster than the typical doubling time for bacteria cells (∼30 min to 1 h or longer, depending on growth conditions). After segregation, the positions of the PCs become largely localized regardless of the elapsed time (Fig. 3 *B*). Generally speaking, directed segregation provides a mean of segregation followed by locking in the positions of the sister PCs before cell division. Fig. 4 *B* suggests that a segregation distance of ∼1/2 the nucleoid length always ensures segregation fidelity, regardless of the initial positions of the PCs (curve a). However, when the segregation distance is shorter than half the nucleoid length, the partition fidelity will be susceptible to the initial positions of the PCs. And in reality, the initial positions of PC splitting would vary between the middle of the nucleoid and the poles (15). Therefore, when the initial positions of the PCs are not at mid-nucleoid, a separation distance shorter than half the nucleoid length will decrease the partition fidelity (Fig. 4 *B*, *curves b* and *c*). On the other hand, plasmid segregation fidelity requires that this separation distance cannot be too large. Otherwise, when the sister PCs are initially at mid-nucleoid, one of the PCs can reach its respective poles but then move back, resulting in pole-to-pole oscillation, which introduces large uncertainties in segregation efficiency, as suggested by Fig. 4 *A*. To achieve robust segregation for all possible initial positions, we therefore suggest that half the nucleoid length is the optimal separation distance for faithful plasmid partitioning, independent of the initial positions of the PCs. This optimal separation distance requires that ParA-mediated directed segregation operates in a specific parameter regime, close to, but not in, the pole-to-pole oscillation zone in Fig. 3 *A*, where the partition fidelity is ∼100% (Fig. 4 *A*).

As the average position of the parental PC is the middle of the nucleoid, this optimal separation distance targets daughter PCs to the quarter positions, which agrees with the observed regular positioning of plasmids (8, 9, 10, 11, 12, 13, 14, 15). An important note is that these quarter positions will naturally become the mid-nucleoid positions for the daughter cells when the mother divides. Our model thus suggests that the alternative pattern of PC positioning between mother and daughter cells reflects an optimal control mechanism for ensuring plasmid partition fidelity. Also, as the directed-segregation regime that confers high segregation fidelity is close to the pole-to-pole oscillation regime in the phase diagram (Fig. 3 *A*), fluctuations in some of the parameters, such as ParA and ParB concentrations, or plasmid copy number, could occasionally push the system into the pole-to-pole oscillation regime. This suggests that a minor fraction of cells would display pole-to-pole oscillations of two plasmids, whose partition is accordingly not robust. We therefore suggest that the observed pole-to-pole oscillation of plasmids, although infrequent, could be indicative of a fluctuation from the underlying mechanism of directed segregation for optimal partitioning. This is consistent with the observation that such oscillation only occurs in ∼1% of cells (11).

### Partition efficiency is impacted by the PC copy number and nucleoid length

The above results suggest that the ParA-type partition system might operate in the regime of directed segregation of two PCs at approximately a half nucleoid-length apart, as this distance provides optimal partition fidelity. To further test this notion, we next examine the response of the directed-segregation mode to perturbations. Specifically, what happens if there are more than two PCs? And how will this optimal scheme play out when the nucleoid length changes?

**Figure 5:**
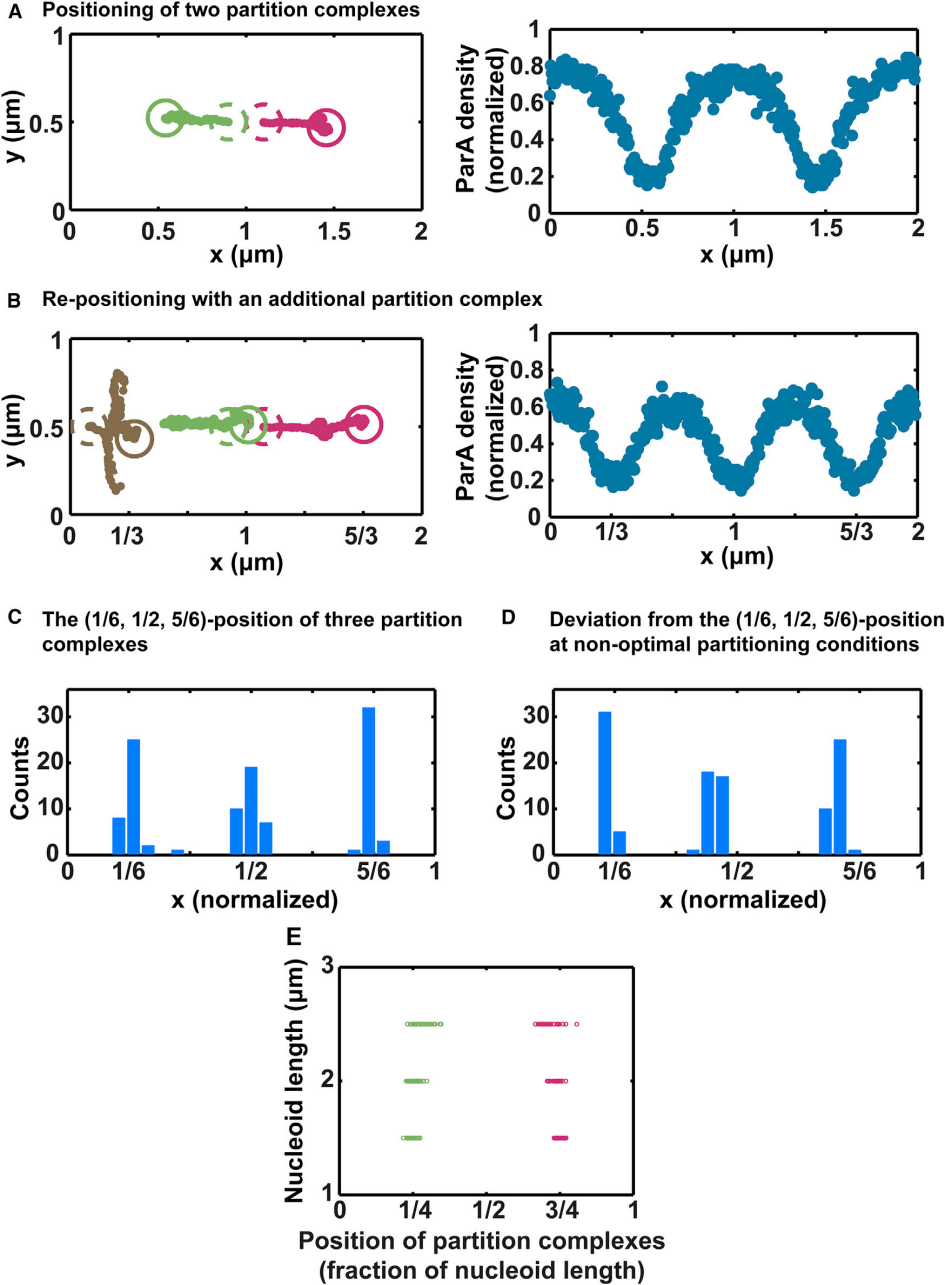
Self-correcting PC positioning. (*A*) Steady-state positions of two PCs. The two PCs (*dashed green* and *magenta circles*) initially position at the mid-nucleoid, then undergo directed segregation for 10 min, and end up around steady-state positions (*solid green* and *magenta circles*). *Left*: A typical trajectory of two PCs before the introduction of the third PC. *Right*: Corresponding symmetric distribution of ParA concentration with local minima surrounding the plasmids. (*B*) PC re-positioning over 10 minutes after introducing an additional PC (*brown*) into the two-PC system (*green* and *magenta*). (*C*) Histogram of the positions of the three PCs in (*B*) over the 10-min period after introducing the third PC. For (*A*)–(*C*), the parameter sets are the same, corresponding to *k*_a_ = 0.047/s and *k*_off_^0^ = 2.0/s in the phase diagram of Fig. 3*A*, which yield a separation distance 0.673 *μ*m. (*D*) Equidistant PC spacing is robust when the overall positioning of the PCs shifts along the long axis of the nucleoid. In this example, the parameter set is *k*_a_ = 0.06/s and *k*_off_^0^ = 2.0/s, which yield the separation distance 0.637 *μ*m. (*E*) Predicted optimal PC positioning as a function of nucleoid length. Here, the two PCs are initially at mid-nucleoid. In addition, to achieve separation distance increase with nucleoid length, we vary the parameters in the phase diagram of Fig. 3*A*. While keeping the ParA·ATP-ParB bond dissociation rate fixed at *k*_off_^0^ = 1.2/s, we change the ParA replenishment rate from *k*_a_ = 0.02/s to *k*_a_ = 0.019/s and 0.018/s, to increase the average separation distance from 0.751 to 0.954 and 1.076 *μ*m, corresponding to the cases with nucleoid lengths of 1.5, 2.0, and 2.5 *μ*m, respectively.

Our simulations show that when an additional PC is introduced to the model simulation with two PCs in the directed-segregation mode, the relative positions of the individual PCs adjust to establish equidistant positioning among the three PCs (Fig. 5, *A* and *B*). Accompanying this adjustment, the corresponding ParA spatial distribution evolves and establishes a symmetric distribution surrounding each focus (Fig. 5 *B*). While maintaining an equidistant relationship, the overall locations of the three foci can shift (Fig. 5, *C* and *D*). This model result remarkably recapitulates the experimental observations of the in vivo ParA-mediated positioning of multiple P1 or F plasmids (7, 8, 9, 10, 11, 12, 13, 14, 15) and other large organelles such as carboxysomes that utilize the same partition machinery for positioning along the nucleoid (39). Because faithful plasmid segregation only needs each daughter cell to inherit one copy of the plasmid, an equidistant configuration per se may not have physiological significance. Rather, it could reflect a natural consequence of the underlying mechanism that is principally optimized for faithful segregation of two PCs.

This model result leads to an important point: if the ParA-type system is optimized for plasmid partitioning for all possible initial positions of plasmid replicas sharing the same nucleoid, then the model predicts that the separation distance between the two PCs should adapt to nucleoid length such that their ratio remains ∼1:2. For instance, for PCs splitting from the middle of the nucleoid, the model predicts that optimization of directed segregation would on average position them around the quarter positions regardless of nucleoid length (Fig. 5 *E*). In the model, this required longer separation distance entails a slower ParA replenishment rate and/or a faster ParA-ParB bond dissociation rate (Fig. 3 *D*). We note that the simulation domain represents a nucleoid, not the entire cell. The positions of PCs in the model are relative to the nucleoid frame, whereas most of the relevant experimental measurements refer to PC positions in the cell frame. Experiments suggest that nucleoid length is largely proportional to cell length (40), the exact ratio of which could depend on species and experimental conditions. If the nucleoid only fills a fraction of cell length and/or the nucleoid center does not overlap with mid-cell, then the predicted PC positions will shift in the cell frame according to the geometry consideration. On the other hand, if the nucleoid fills most of the cell length, then our predicted relative positions of PCs along the nucleoid long axis could roughly reflect the corresponding positions in the cell frame. In this regard, the existing experimental data from different model systems could lend support to our predictions. Indeed, as predicted by the model (Fig. 5 *E*), independent of cell length, for cells with two PCs, they are centered around the quarter-cell positions (7, 8, 9, 10, 11, 12, 13, 14, 15). This observed size invariance suggests that the ParA-type system is optimized for faithful plasmid partition.

## Discussion

We report a Brownian ratchet mechanism mediated by the ParA-type system that directs persistent movements of PCs necessary for proper segregation of low-copy-number plasmids. Our model can recapitulate the full spectrum of PC motility modes observed in vivo. According to our model, the directed-segregation mode of daughter PC movement, combined with a characteristic separation distance of half the nucleoid length, results in optimal plasmid partition efficiency. The experimentally observed regular spacing between multiple PCs was also reproduced by the model, which reflects the underlying partitioning mechanism.

In comparison with the earlier models, our model elements provide important additional insights into the mechanistic underpinnings and functional implications of ParA-mediated partitioning. Conceptually, the reaction-diffusion models (11, 34), the chemophoretic model (41), and our model all hinge on the ParA concentration gradient in front of the cargo, which effectively presents an attractive potential that drives the cargo forward. That is, all these models support directed movement with the same underlying mechanism. ParA-driven cargos move on a 2-D substrate. The cargos alone typically undergo rapid 2-D diffusion, yet the corresponding ParA-mediated movement trajectories are quite straight (21). The reaction-diffusion models, however, are essentially 1-D (e.g., (11)). Hence, it is difficult to extend the results of these models to the 2-D case. Indeed, we showed in previous modeling efforts that a 2-D reaction-diffusion model with a ParA gradient in front of the cargo cannot support straight movement trajectories unless the intrinsic cargo diffusion is significantly slowed down to levels well below the measured values of the diffusion constant (42). The reason for this lack of persistency is that with a driving force in only one direction, nothing restricts diffusive excursions in the orthogonal directions in 2-D; consequently, the resulting movement will display significant lateral excursions, rather than a straight trajectory (22). In contrast, our model is an agent-based model that describes the dynamics of individual bonds between the ParB-laden cargo and the ParA-covered 2-D substrate. Importantly, our model supports directed and persistent movement in two dimensions ((22) and this work), whereas the other models cannot. The key mechanistic insight from our model is that the transient tethering arising from the ParA-ParB contacts collectively drives forward movement of the cargo and quenches diffusive motion in orthogonal directions. From a functional perspective, to our knowledge, our model is the first to recapitulate all observed plasmid motility patterns, whereas other models have focused exclusively on a subset of these patterns, in particular, pole-to-pole oscillation (e.g., (8, 11, 43)). Whereas pole-to-pole oscillation is an interesting phenomenon, we show that this motility mode compromises plasmid partition fidelity (Fig. 4 *A*). We further show that it is the directed-segregation mode of motility that maximizes partition fidelity (Fig. 4 *A*). Our finding provides an explanation for the experimental observation that only 1% of cells display pole-to-pole oscillation whereas replicated plasmids in the majority of cells undergo directed segregation (11). Thus, our model provides a functional perspective on different plasmid motility patterns.

Directed segregation as a reliable mechanism for plasmid partition or positioning could have its limits. First, the model suggests that PC motility hinges on the physical size of the cargo (Fig. S2). A unifying feature of bacterial cargos that utilize ParA-mediated intracellular organization (14, 44, 45), such as large plasmids, chromosomes, carboxysomes, and chemotaxis clusters, is that all are massive macromolecular bodies in the cell. If the PCs were smaller, they would tend to undergo more diffusive movement (Fig. S2) due to the faster intrinsic diffusion and fewer ParA-ParB bonds, which cannot provide sufficient tethering to quench cargo diffusion. Larger cargos are thus better suited for robust partitioning by this mechanism (Fig. S2). This perspective also pertains to the necessity of clustering plasmid copies into a smaller number of PCs during segregation, which increases the effective size of the cargo and hence partition fidelity.

Second, the model suggests an interesting dependence of ParA-mediated positioning on the number of PCs (Fig. S6). As the number of PCs in the system increases, the ParA on the nucleoid, and hence the number of ParA-ParB bonds, become more and more scarce, due to the depletion effect exerted by PCs. We showed that with a sufficient amount of ParA, the model could still drive directed movements with increasing numbers of PCs. However, as the number of PCs increases, the space along the long axis becomes limited, and the PCs tend to utilize the space along the short axis as well. Consequently, these PCs can still achieve equidistant configuration; it’s just that they do not align along the long axis. On the other hand, if there is insufficient ParA, then the nucleoid-bound ParA could be depleted by the PC-bound ParB to such an extent that the remaining ParA-ParB bond-mediated tethering becomes insufficient to fix the positions of these PCs. As a result, similar to the case of random diffusion/pole-to-pole oscillation (Fig. 4 *A*), PC positioning approaches a random distribution (Fig. S6) that could jeopardize the fidelity of partition. In reality, low-copy-number plasmids that depend on ParA-mediated systems rarely generate more than four or five PCs occupying one nucleoid. This brings up an interesting question pertaining to the control mechanism of PC numbers in conjunction with total protein amounts, which we will investigate in the future.

Third, faithful plasmid partitioning described by our simplified ParA-based Brownian ratchet mechanism appears to be limited by nucleoid length. When the nucleoid length increases well beyond its normal range (∼2 *μ*m), our results suggest that there would be increasing uncertainty in targeting the daughter PCs of a single parental PC from mid-cell to the quarter positions (Fig. S7). This is because increasing the separation distance in our mechanism requires slowing down the refilling of the ParA-depletion zone and/or speeding up the ParA-ParB bond dissociation (Fig. 3, *A* and *D*). Either way, this decreases the number of ParA-ParB bonds and hence weakens the associated tethering such that random motions begin to manifest more strongly. This drives the system from the directed-segregation regime toward the pole-to-pole oscillation and diffusion regimes, resulting in more and more uncertainties in plasmid positioning and segregation fidelity (Figs. 4 *A* and S5).

This finding provides an interesting perspective on the observed positive correlation between PC number and nucleoid size. The rich media that promote bacterial cell growth favor both nucleoid growth and plasmid replication. Consequently, the plasmid DNA replication rate and resulting plasmid copy number would parallel the nucleoid/cell size. That is, as the nucleoid elongates as chromosomal DNA replicates, there often are more than two PCs associated with a nucleoid. We suspect that the PC split/fusion dynamics could have a homeostatic control mechanism to balance nucleoid size with the associated number of PCs via plasmid DNA clustering dynamics. In this context, although the homeostatic control mechanism of plasmid clustering is not well understood, it likely plays a role in ensuring the fidelity of plasmid partition function. Therefore, a more comprehensive appreciation of ParA-type plasmid partition system requires a better mechanistic understanding of plasmid clustering.

We note that although the same ParA-type partition system drives bacterial chromosome segregation, it is an open question whether the same Brownian-ratchet mechanism is at play. Unlike plasmid segregation, in which the nucleoid is a separate structure that the PC can “walk” along, chromosome replication and segregation are concurrent processes in bacteria (46, 47, 48). The segregating chromosome essentially “walks” along the nucleoid that is undergoing drastic remodeling itself, as the chromosome is the major structural component of the nucleoid. In other words, the cargo is also part of the substrate. Currently, it is unclear how to properly characterize the motion of the PC in this context. The chromosome will probably slow down the effective diffusion of the PC, which is further constrained by ParA-ParB bond tethering, as shown by our model (22). Whereas the details of chromosome segregation remain uncertain, conflating the measured in vivo diffusion constant of the PC with the intrinsic diffusion constant for modeling purposes (20) leads to an underestimate of the rate of directed PC motion and to the erroneous claim that the Brownian ratchet mechanism is too slow to account for the observed chromosome segregation (20). Nevertheless, it remains to be seen whether and how the Par-system-mediated Brownian ratchet mechanism plays out in bacterial chromosome segregation, which is the topic of our future investigation.

Our model necessarily makes several simplifying assumptions, and several aspects could be further refined. ParAB*S* biochemistry is almost certainly more complicated than our current model scheme (49). We have not explicitly defined the steps involved in reactivation of ParA in the cytosol after ATP hydrolysis or exactly how the energy from ParA ATP hydrolysis is linked to cargo movement. Moreover, we have not evaluated the impact of the weak, but possibly significant, non-specific binding of ParB on the nucleoid or the effect of binding and turnover of ParA on the PC in this simulation. Although we believe the qualitative aspects of the outcome of this study are solid, quantitative aspects of the PC dynamics prediction could certainly be influenced by these details of the biochemical steps that have yet to be addressed and will be the topic of our future work.

From the standpoint of mechanics, our model makes the simplifying assumption that plasmid partitioning can be accurately described as a 2-D system: both the nucleoid and the PC are treated as rigid, impenetrable 2-D objects, between which ParA and ParB molecules can bind to form elastic bonds bridging the nucleoid and the PC. However, in vivo plasmid partition plays out in a three-dimensional space, wherein neither the nucleoid nor the PC is a rigid, impenetrable body. Rather, they are soft dynamic entities, and protein molecules could diffuse through them. Because of the volume-exclusion effects involving long DNA chains, PCs are not expected to readily diffuse through a nucleoid, but a certain degree of partial inter-penetrations would be expected (27). On one hand, these details would influence the quantitative evaluation of our model. Construction of such a more realistic three-dimensional modeling system will have to wait for future in vivo experiments that can further characterize the dynamic and mechanical properties of the nucleoid and PCs. By the same token, the nucleoid is a dynamic ultra-structure with a topography that undergoes constant remodeling (50). Whereas nucleoid elongation and segregation are typically slow compared to the rate of low-copy-number plasmid replication and partitioning (3), its dynamical remodeling could potentially introduce another layer of complexity to plasmid partitioning. Understanding how plasmid segregation is influenced by nucleoid duplication and resolution into two nucleoids will be the subject of future study. Furthermore, plasmids that harbor the same or very similar partition machinery can be incompatible, i.e., co-resident plasmids negatively affect the inheritance of at least one of them (51). One of our future efforts will be to leverage our model to shed light on the nature of plasmid incompatibility.

## Author Contributions

L.H., A.G.V., K.M., K.C.N., and J.L. designed the research, analyzed the data, and wrote the article. L.H. performed the research.

## Supporting Material

Document S1. Supporting Materials and Methods, Figs. S1–S7, and Table S1

Document S2. Article plus Supporting Material
