# Supplementary material for: Brownian Ratchet Mechanism for Faithful Segregation of Low-Copy-Number Plasmids
Source: Biophys J. 2017 Apr 11;112(7):1489–502. doi: 10.1016/j.bpj.2017.02.039 (PMC5390091; doi:10.1016/j.bpj.2017.02.039)
Supplement: Document S1. Supporting Materials and Methods, Figs. S1–S7, and Table S1 [file mmc1.pdf]

Biophysical Journal, Volume *112*

Supplemental Information

Brownian Ratchet Mechanism for Faithful Segregation of Low-Copy-Number Plasmids

Longhua Hu, Anthony G. Vecchiarelli, Kiyoshi Mizuuchi, Keir C. Neuman, and Jian Liu

Section 1. Table S1. Model parameter table

Parameter	Physical meaning	Value, units	Reference
k_s	Spring constant of ParA·ATP-ParB bond (*)	0.05 pN/nm	(1)
k_{on}	Association rate of ParA·ATP-ParB bond (*)	3.3*10^3/sec	(2)
k_{off}^0	Intrinsic rate of ParA·ATP-ParB bond dissociation (*)	0.5 – 3.0/sec	(2)
k_a	Rate of cytosolic ParA·ATP refilling to substrate	0.01 – 0.1/s	(3-10)
$k_{d,T}$	Intrinsic rate of ParA·ATP dissociation from substrate	0.01/s	(5, 6)
$k_{d,D}$	Intrinsic rate of ParA* dissociation from substrate	5.0/s	(5, 6)
D_p	Diffusion coefficient of plasmid focus	10^5 nm^2/s	(11)
D_T	Lateral diffusion coefficient of ParA·ATP along substrate	1250 nm^2/s	(6)
D_D	Lateral diffusion coefficient of ParA* along substrate	1250 nm^2/s	(6)
R	Radius of plasmid focus	100 nm	(12, 13)
ρ_B	Density of ParB on the disk	~0.05/nm^2	(4, 12)
L_e	Equilibrium length of the ParA·ATP-ParB bond (*)	50 nm	This article
L_a	Maximal bond length for newly formed bonds (*)	53 nm	This article
X_c	Threshold bond length extension of existing bonds (*)	10 nm	(2)
f_c	Characteristic bond dissociation force (*)	0.8 pN	(2)

(*) Note that, the ParA·ATP-ParB bond here refers to a series of connected tethering that bridges between the plasmid and nucleoid DNA, *i.e.*, nucleoid DNA tether–ParA·ATP–ParB–plasmid.

Section 2. Model parameter consideration or estimation

Association and dissociation rates of ParA·ATP-ParB bond (k_{on} and k_{off}^0)

Compared with their values in our previous study pertaining to *in vitro* conditions (2), both k_{on} and k_{off}^0 are decreased 3 fold in the current study to account for the much slower speed of the directed cargo movement *in vivo*. While there is no direct

experimental measurements to substantiate these parameter values, changing them will not affect the qualitative essence of the conclusions, as demonstrated by phase diagrams presented in this, and our previous, work (2).

Rate of ParA·ATP replenishment onto substrate (k_a)

Because the model describes the cytosolic ParA·ATP dimer only with its overall concentration, the ParA refilling rate reflects a combination of two effects. First, at the single molecule level, it takes minutes for the ParA·ADP dissociated from DNA carpet to reset its DNA-binding ability (3). Once "primed", the subsequent DNA-binding step of the ParA·ATP dimer is very fast, on the order of 20 ms (6). Consequently, the rate-limiting step is priming the ParA·ATP dimer, which sets the lower bound of the ParA refilling rate ~ 1/10min = 0.0016/sec. On an ensemble level of cytoplasmic ParA, however, there is always a certain fraction that has already re-gained DNA-binding ability. As cytoplasmic ParAs are small molecules and hence expected to diffuse rapidly in the three-dimensional volume between the nucleoid and cell membrane, this pool of "primed" ParA·ATP dimers can readily rebind to vacant sites on DNA carpet. This refilling process should be faster than that from single-molecule level. If it is not limited by ParA diffusion in cytoplasm, then this ParA refilling rate is ~ 1/(20 ms) = 50/sec. In reality, the ParA concentration is always limited. Given that the concentration of ParA molecules *in vivo* is ~ micromolar, *i.e.*, ~ 1000s of ParA per cell (4, 7, 8), most of which is DNA-bound (9, 10), the number of ParA in the cytoplasm is expected to be on the order of 100s. We assume on the order of 200 ParA molecules. In the default model setup, where the nucleoid domain is a 2 μm by 1 μm rectangle and 5 nm lattice site spacing, there are ~80000 binding sites on the nucleoid surface. Note that this ParA density is still less than the estimated ParA saturation density on the nucleoid (5, 6). The probability that each site has a juxtaposed ParA molecule "hovering" above that is ready to rebind is ~ 200/80000 = 1/400. This reduces the effective refilling rate from 50/sec to 0.13/sec. Additionally, *in vitro* FRAP measurements show that in the absence of SopB, there are two apparent rates of SopA exchanging from nonspecific DNA: 4.6/min and 0.6/min (6), which presumably reflects two populations of SopA molecules with different conformations. This gives a range of ParA refilling rate to be 0.01 – 0.08/sec. Certainly the actual ParA refilling rate depends on the concentration of cytoplasmic ParA. Taken together, we estimate that the effective ParA refilling rate in our model, k_a, is 0.0016/sec – 0.13/sec. In our simulation, the default value of ParA refilling rate is chosen to be 0.05/sec. We note that the balance between this dissociation rate and the ParA refilling rate dictates the overall steady state concentration of nucleoid surface-bound ParA. In this sense, changing either $k_{d,T}$ or k_a modulates the ParA coverage. By keeping $k_{d,T}$ = 0.01/sec while varying the ParA refilling rate k_a, we can explore the full range of ParA coverage that distinctively affects the plasmid movement (Figures 2 and 3).

Intrinsic rate of ParA·ATP dissociation from substrate ($k_{d,T}$)

In vitro experiments show that the rate of SopA·ATP dissociation from DNA carpet is 1.9/min in the absence of SopB (6). A similar value is measured for ParA·ATP as well, which ranges over 0.008 – 0.03/sec (5). The model default value for the intrinsic rate of ParA·ATP dissociation from substrate is $k_{d,T}$ = 0.01/sec. As elaborated above, the ratio between this rate and the ParA refilling rate k_a modulates the overall coverage of ParA on the DNA carpet, which has been explored in Figures 2 and 3.

Rate of ParA* dissociation from the substrate ($k_{d,D}$)

ParB is known to stimulate ParA dissociation from DNA carpet (5, 6). The measured apparent rate of ParB-stimulated ParA dissociation or SopB-stimulated SopA dissociation from DNA carpet range from 0.008/sec to more than 1/sec depending on conditions (5, 6). This process corresponds to the two consecutive steps in the model, i.e., the ParA·ATP–ParB bond dissociation (as above) and the subsequent ParA* dissociation from the substrate. Therefore, the combined rate of these two steps should correspond to this measured rate. The model assumes a default rate of ParA* dissociation from the substrate, $k_{d,D}$, of 5.0/sec. Combined with the default rate of ParA·ATP–ParB bond dissociation (k_{off}^0 = 1/sec), the overall rate of ParB-stimulated ParA dissociation from DNA carpet is $1/(1/k_{d,D}+1/k_{off}^0)$ ~ 0.8/sec, which is within the measured range. Importantly, our model results show that the rate of ParA* dissociation from DNA carpet needs to be sufficiently fast to yield a discernible ParA depletion zone, which is necessary for directed plasmid movements. We speculate that energy from ATP hydrolysis renders the ParB-stimulated ParA–DNA dissociation irreversible. As a result, a combination of the mechanical actions of ParA-ParB bonds and ParA ATP hydrolysis creates and maintains the ParA-depletion zone, which underlies the core of the ratchet mechanism.

Diffusion coefficient of plasmid focus D_p

The plasmid focus diffusion coefficient refers to lateral diffusion on the DNA surface without any specific interactions. *In vitro* reconstitution experiments (11) show that the lateral diffusion coefficient for a microbead of 1 μm diameter is ~ 0.1 μm^2/sec. We use this value as a reference point to estimate the diffusion coefficient of plasmid focus. The default diameter of the plasmid focus in our model is 200 nm, which is elaborated separately in a later section. Whereas this smaller size is expected to increase the lateral diffusion by ~ 5 fold, the cytoplasm is 5-10 - fold more viscous than water, which counteracts the effect of the reduced size on the diffusion constant. Based on these considerations, we choose the diffusion coefficient of the plasmid focus ~ 0.1 μm^2/sec. Varying it does not fundamentally alter the key conclusions of the model, although quantitative aspects of the results change. A final note on the value of the intrinsic diffusion coefficient: tethering by ParA·ATP–ParB bonds reduces the mobility of the plasmid focus by orders-of-magnitude, resulting in a much smaller apparent diffusion coefficient as measured in experiments (14).

Effective diffusion coefficient of ParA on the substrate (D_T and D_D)

As the model simulation is carried out on a lattice, we need to convert the diffusion coefficient measured in experiments to that used in our simulations accordingly. In the model, the effective diffusion coefficient of ParA on the substrate takes into account two consecutive processes: ParA hopping along the substrate followed by binding to the nonspecific DNA carpet. The measured apparent diffusion coefficient of ParA on the nonspecific DNA carpet is ~ 0.85±0.14 $\mu m^2/s$ (6). It takes ~ 25 – 35 μs for a particle to diffuse over a 5 nm-range, which is much shorter than the ParA·ATP–DNA binding time of ~ 20 ms (3). Thus, diffusion is limited by the ParA·ATP binding to DNA, which leads to an effective lateral diffusion coefficient of ParA·ATP as ~ 1000 – 1500 nm^2/s in our simulation. We choose the default value of the lateral diffusion coefficient of ParA·ATP to be 1250 nm^2/s. For simplicity, the model keeps the lateral diffusion coefficient of ParA* the same as that of ParA·ATP. Changing these diffusion coefficients within a reasonable range does not critically affect the model results.

Radius of plasmid focus R

Recent super-resolution experiments in *E. coli* demonstrate that the confinement region of ParB/*ParS* complex in one focus is 100 – 300 nm (12), which we assume to define the size of a plasmid focus. Similarly, the diameter of ParB/*ParS* complexes focus on chromosomal DNA in *C. crescentus* is shown to be ~ 150 nm (13). In our model, the default value of plasmid focus radius, R, is thus chosen to be 100 nm. Varying the size of plasmid focus is predicted to influence the motility mode by shifting the phase boundaries in the phase diagram (Figure S3), whereas the essential features of the model are largely preserved.

Density of ParB in plasmid focus

PALM studies in *E. coli.* show that more than 90% of ParB dimers is confined in an area of 100 – 300 nm in diameter (12). Given that there are ~ 800 ParB dimers in this *E. coli* system (4), this suggests a ParB dimer density in plasmid focus of ~ 0.01 – 0.09 ParB dimer/nm^2. We choose the nominal density of ParB dimers to be ~ 0.05 ParB dimer/nm^2.

Spring constant of ParA·ATP-ParB bond (k_S)

In the model, the ParA·ATP–ParB bond describes the connection between the plasmid focus and the nucleoid surface. As a result, the effective bond stiffness reflects the combined elasticity of a series of connected springs: nucleoid–ParA·ATP–ParB–plasmid. Because both ParA and ParB are very small proteins, it is expected that the proteins themselves are very stiff. Moreover, the binding affinity between ParA·ATP and ParB gives a rough sense that their bond potential energy is > 10 k_BT. Given that the

typical dimension pertaining to ParA·ATP–ParB is only several nm, the effective stiffness of this bond is expected to be ~ pN/nm. In addition, ParB spreading and bridging are believed to collapse the plasmid into a focus. Thus, the ParB *cis*-interactions and the interaction between ParB and *ParS* together define the effective stiffness of ParB–plasmid bond, which is expected to be comparable to that of the ParA·ATP–ParB bond. The last part of the series of the connected springs is the nucleoid–ParA·ATP bond, the elasticity of which largely depends on the deformability of the nucleoid. Because we focus only on the scenario in which plasmid segregation is much faster than nucleoid growth and replication, the model describes the nucleoid as a relatively static object. The nucleoid elasticity in our model therefore refers to its intrinsic mechanical properties without the influence of active remodeling processes that consume ATP. In this regard, the nucleoid elasticity could be inferred from the position fluctuations of chromosomal loci (σ); $\frac{1}{2}k_N\sigma^2 \sim k_BT$, where k_N is the elastic spring constant of the nucleoid. In fixed cells, the position fluctuations of chromosomal loci (σ^2) are measured at different time lags (1). Given that the typical lifetime of a ParA·ATP–ParB bond is ~ 1 sec, the position fluctuations of chromosomal loci (σ^2) at 1 sec is the most relevant, which is ~ (1 – 2) $\times$ 10^{-4} μm^2 (1). Since this value is obtained from fixed cell, it reflects ambient thermal fluctuations for which the relation $\frac{1}{2}k_N\sigma^2 \sim k_BT$ can be applied. Therefore, the spring constant of nucleoid in the context of our model is ~ 0.04 – 0.08 pN/nm, which is the softest spring in the nucleoid–ParA·ATP–ParB–plasmid tether. This suggests that the deformation of the ParA·ATP–ParB bond in the model effectively reflects the intrinsic nucleoid elasticity. While variation of the spring constant within this range does not affect the model essence, we choose the default spring constant of the effective ParA·ATP–ParB bond k_s as 0.05 pN/nm.

Characteristic lengths pertaining to ParA•ATP-ParB bond (L_e, L_a, and X_C)

The nucleoid is in close contact with cell periphery, which leaves a small gap between the nucleoid surface and the cell membrane. For instance, Fisher *et al*. show that whereas the diameter of *E. coli* is only 0.6 μm, the width of the nucleoid is ~ 0.48 μm (15). The distance between the nucleoid surface and cell membrane is thus ~ 60 nm. Given that the plasmid focus itself will occupy some fraction of this gap, the remaining space is expected to be on the order of several tens of nm, which sets a physical constraint on the ParA·ATP–ParB bond length. In the model, we use 50 nm as the equilibrium bond length (L_e), 53 nm as the maximal bond length for newly formed bonds (L_a), and 60 nm as the maximal bond length before instantaneous bond dissociation (L_e+X_C). We note that the absolute values of L_e, L_a, and L_e+X_C are not critical. Rather, it is the relative differences between these bond lengths that underlie a key aspect of the Brownian ratchet mechanism; namely, the newly formed bonds are pre-stretched by thermal energy, which subsequently drives the cargo movement. Therefore, the Brownian ratchet aspect of the model persists as long as (L_a - L_e) is less than X_C; in other words, the maximum bond extension is larger than the bond pre-stretching during bond formation. This is a necessary condition of an efficient Brownian ratchet

mechanism, which has been systematically demonstrated in our previous work (2). In our current paper, the relative differences between L_e, L_a, and L_e+X_C are kept the same as in the earlier studies corresponding to the *in vitro* conditions (2).

Reference

1. Javer A*, et al.* (2013) Short-time movement of E. coli chromosomal loci depends on coordinate and subcellular localization. *Nature Communications* 4:3003.
2. Hu L, Vecchiarelli AG, Mizuuchi K, Neuman KC, & Liu J (2015) Directed and persistent movement arises from mechanochemistry of the ParA/ParB system. *Proceedings of the National Academy of Sciences* 112:E7055.
3. Vecchiarelli AG*, et al.* (2010) ATP control fo dynamic P1 ParA-DNA interactions: a key role for the nucleoid in plasmid partition. *Molecular Microbiology* 78(1):78-91.
4. Bouet J-Y, Rech J, Egloff S, Biek DP, & Lane D (2005) Probing plasmid partition with centromere-basd incompatibility. *Molecular Microbiology* 55(2):511-525.
5. Hwang LC*, et al.* (2013) ParA-mediated plasmid partition driven by protein pattern self-organization. *The EMBO Journal* 32:1238-1249.
6. Vecchiarelli AG, Hwang LC, & Mizuuchi K (2013) Cell-free study of F plasmid partition provides evidence for cargo transport by a diffusion-ratchet mechanism. *Proceedings of the National Academy of Sciences* 110(15):E1390-E1397.
7. Lioy VS*, et al.* (2015) ParAB partition dynamics in Firmicutes: nucleoid bound ParA captures and tethers ParB-plasmid complexes. *PLos One* 10(7):e0131943.
8. Adachi S, Hori k, & Hiraga S (2006) Subcellular positioning of F plasmid mediated by dynamics localization of SopA and SopB. *Journal of Molecular Biology* 356:850-863.
9. Castaing J-P, Bouet J-Y, & Lane D (2008) F plasmid partition depends on interaction of SopA with non-specific DNA. *Molecular Microbiology* 70(4):1000-1011.
10. Hatano T & Niki H (2010) Partitioning of P1 plasmids by gradual distribution of the ATPase ParA. *Molecular Microbiology* 78(5):1182-1198.
11. Vecchiarelli AG, Neuman KC, & Mizuuchi K (2014) A propagating ATPase gradient drives transport of surface-confined cellular cargo. *Proceedings of the National Academy of Sciences* 111(13):4880-4885.
12. Sanchez A*, et al.* (2015) Stochastic self-assembly of ParB proteins builds the bacterial DNA segregation apparatus. *Cell systems* 1:163-173.
13. Lim H*, et al.* (2014) Evidence for a DNA-relay mechanism in ParABS-mediated chromosome segregation. *eLife* 3:e02758.
14. Reyes-Lamothe R*, et al.* (2014) High-copy bacterial plasmids diffuse in the nucleoid-free space, replicate stochastically and are randomly partitioned at cell division. *Nucleic Acids Research* 42(2):1042-1051.
15. Fisher JK*, et al.* (2013) Four-dimensional imaging of E. coli nucleoid organization and dynamics in living cells. *Cell* 153:882-895.

Section 3. Supplemental Figures

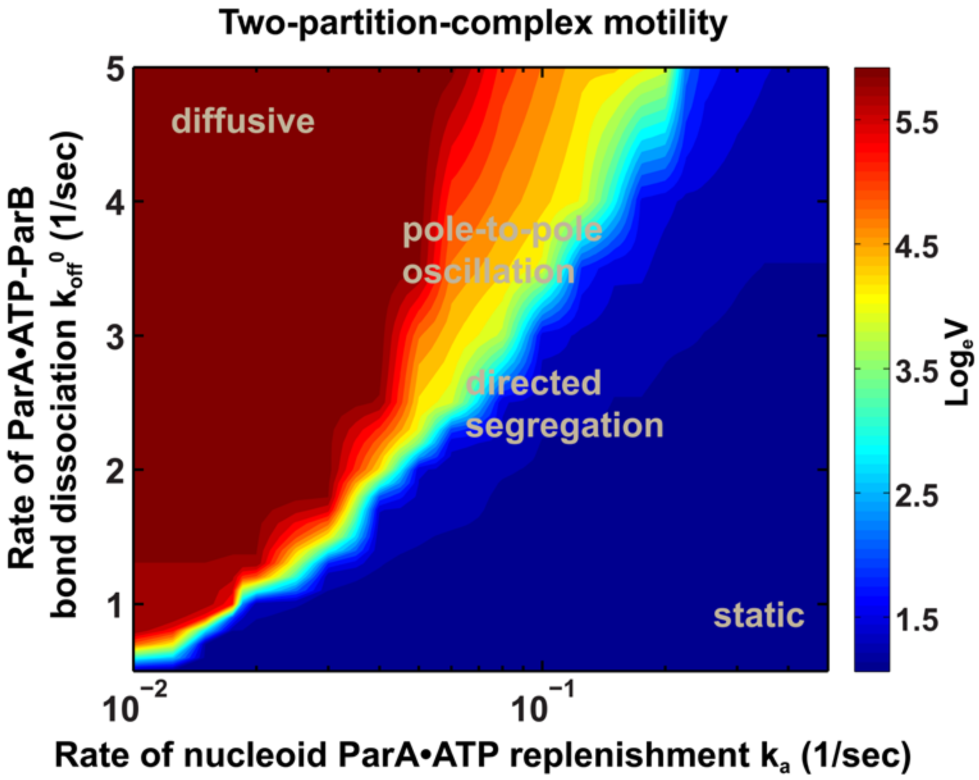

Figure S1. Phase diagram of two-partition-complex motility on a narrower nucleoid. Here, we carry out the same phase diagram study as in Figure 3A, except that the nucleoid width is chosen to be 0.6 μm, instead of 1.0 μm. The color map reflects the log-scale of the average speed of the simulated partition-complex movement over 10 minutes; it is used as a surrogate to represent the gradual transition between different motility patterns. V is the numerical value of the speed in units of nm/sec.

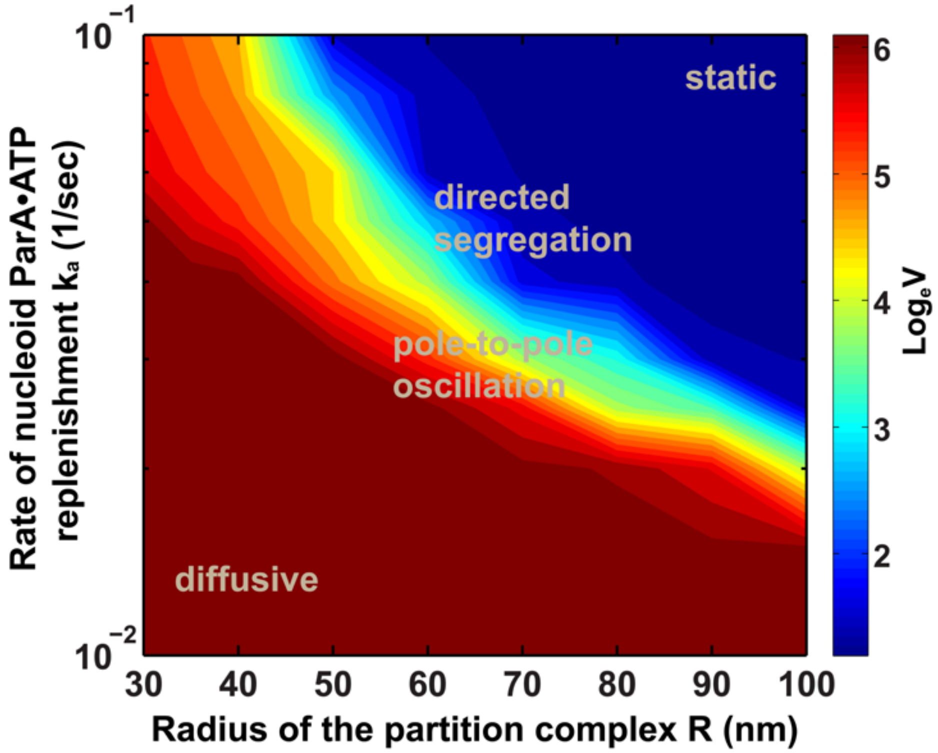

Figure S2. Size-dependence of partition complex motility. In this phase diagram study we simulate the motility of a single partition-complex as its radius and the ParA refilling rate are separately varied, while keeping all other model parameters fixed at their nominal values (see parameter table). The color map reflects the log-scale of the average speed of the simulated partition-complex movement over 10 minutes; it is used as a surrogate to represent the gradual transition between different motility patterns. V is the numerical value of the speed in units of nm/sec.

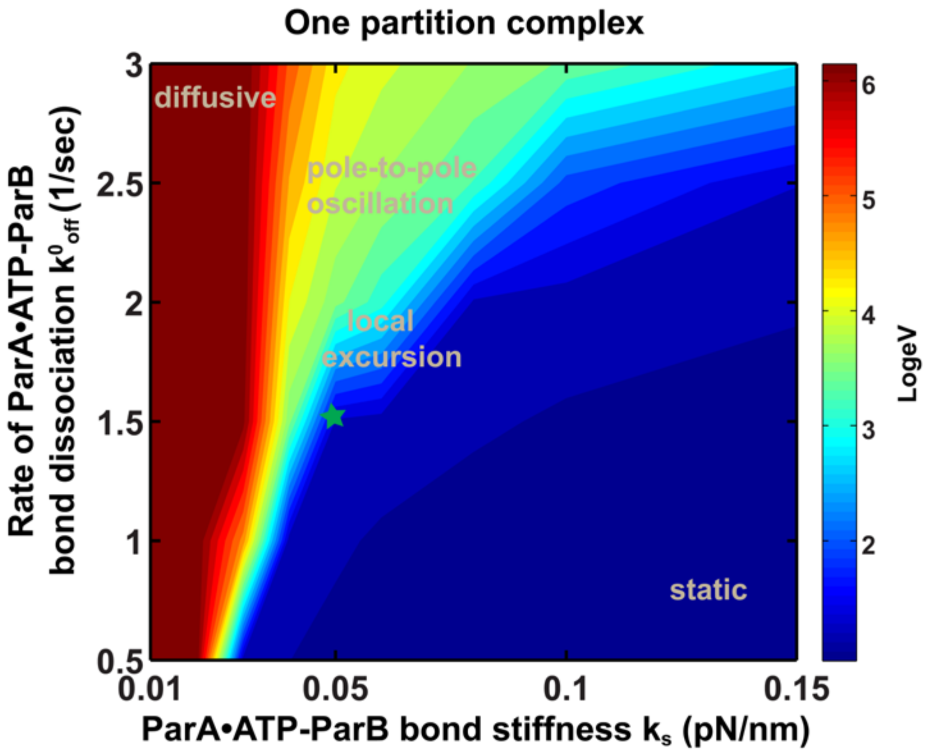

Figure S3. Phase diagram of single PC motility dependence on ParA·ATP–ParB bond stiffness and dissociation rate. The color map reflects the log-scale of the average speed of the simulated partition-complex movement over 10 minutes; it is used as a surrogate to represent the gradual transition between different motility patterns. V is the numerical value of the speed in units of nm/sec.

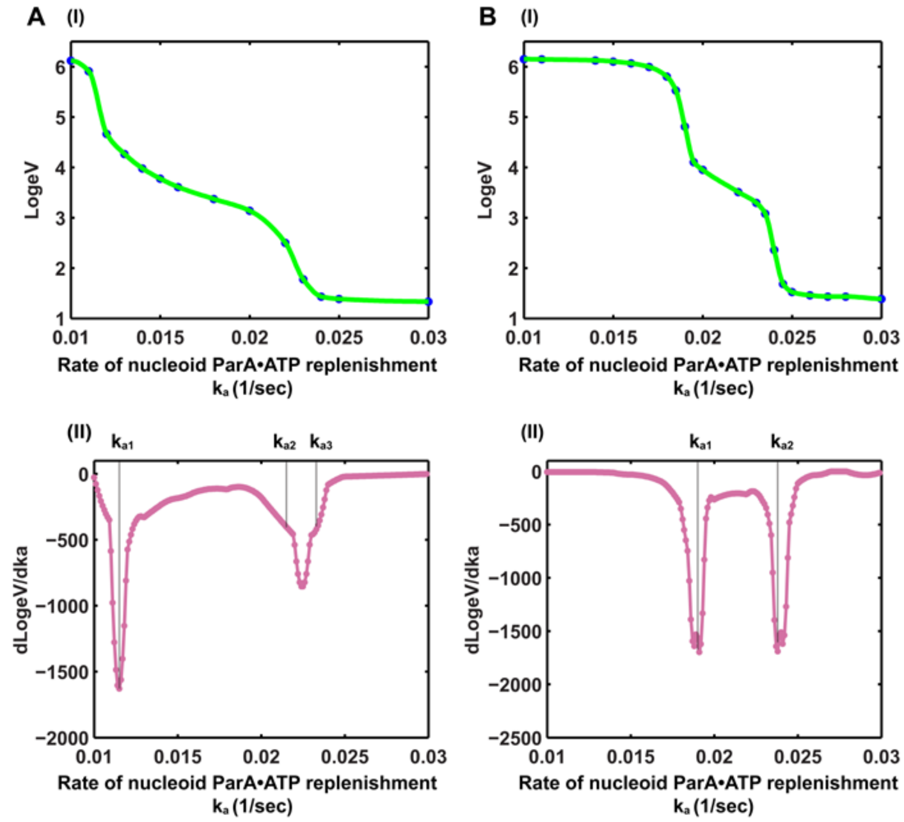

Figure S4. Criteria on characterizing different modes of PC motility using motion speed. (A) Single-PC case. (I) A representative curve showing dependence of PC movement speed V (Log scale) on the ParA refilling rate from cytoplasm k_a. The rest of the model parameters are kept fixed as those in the parameter table. (II) Corresponding slope of the speed vs. k_a curve. This plot shows two peaks separating three distinct phases: diffusion, pole-to-pole oscillation, and being static. From the half height of the peak between pole-to-pole oscillation and being static, we determined two ParA refilling rates, k_a2 and k_a3 that define the range of a transition zone that is important for plasmid segregation. We defined this transition zone as local excursion. On the other hand, the peak between the diffusion and pole-to-pole oscillation modes is very narrow, which is actually narrower than the computation resolution in k_a, 0.001/sec. Additionally, the movement speed of diffusion is in general ill-defined anyway. Rather than pinpointing the exact transition point between pole-to-pole oscillation and diffusion, we did not specify the corresponding transition zone. Instead, for comparison purposes, we approximate the peak position k_a1 as the transition point that delineates pole-to-pole oscillation and diffusion modes. From the $k_\mathrm{a1,}$ k_a2, k_a3, we thus obtained the corresponding movement speeds, V_1 = 204.4 nm/sec, V_2 = 14.6 nm/sec, and V_3 = 5.4 nm/sec, which are the speed criteria used and marked in our dynamic phase diagrams to distinguish different motility modes. (B) Two-PC case. We followed the similar practice as (A). The peak positions k_{a1} and k_{a2} correspond to V_1=104.6 nm/sec and V_2=14.6 nm/sec, respectively. There is no distinct peak separating the directed segregation mode and static movement mode. Instead, a PC is considered to be in the

static mode when the PC on average moves up to 200 nm (the size of the PC) during simulation. The corresponding speed V_3 is about 3.0 nm/sec.

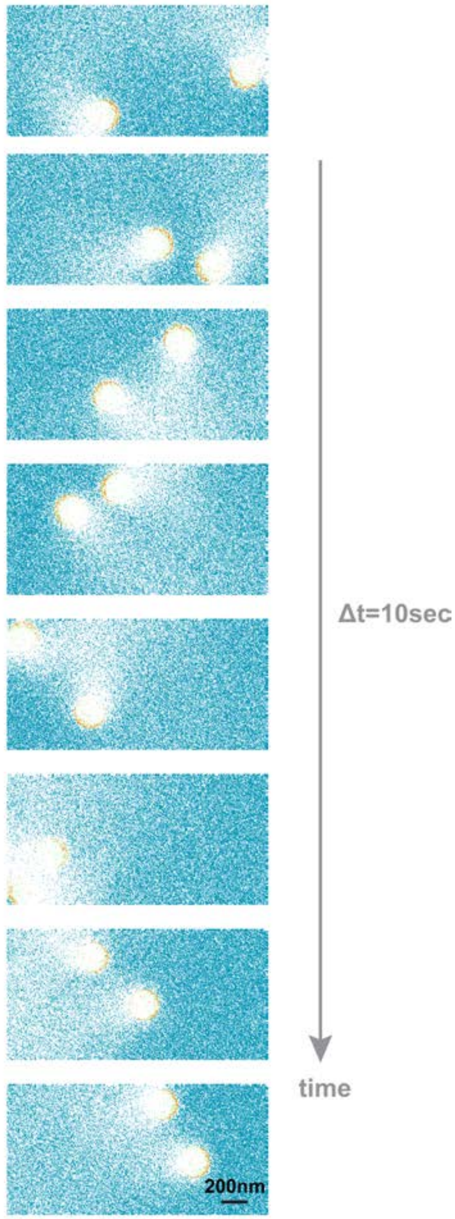

Figure S5. An example trajectory of pole-to-pole oscillation in the two-partition-complex system. The model parameters were k_a=0.035/sec and k_{off}^0 = 2.5/sec in the phase diagram of Figure 3A. The corresponding ParA spatial profile is overlaid with the PC position in each snapshot. ParA·ATP is shown in blue-green, ParA* in green, ParA·ATP–ParB in orange, and the vacant site in white. During their movement, the two partition complexes may be located in the same half or different halves of the nucleoid, resulting in the overall low probability and high variance in segregation probability of partition complex (see Figure 4A).

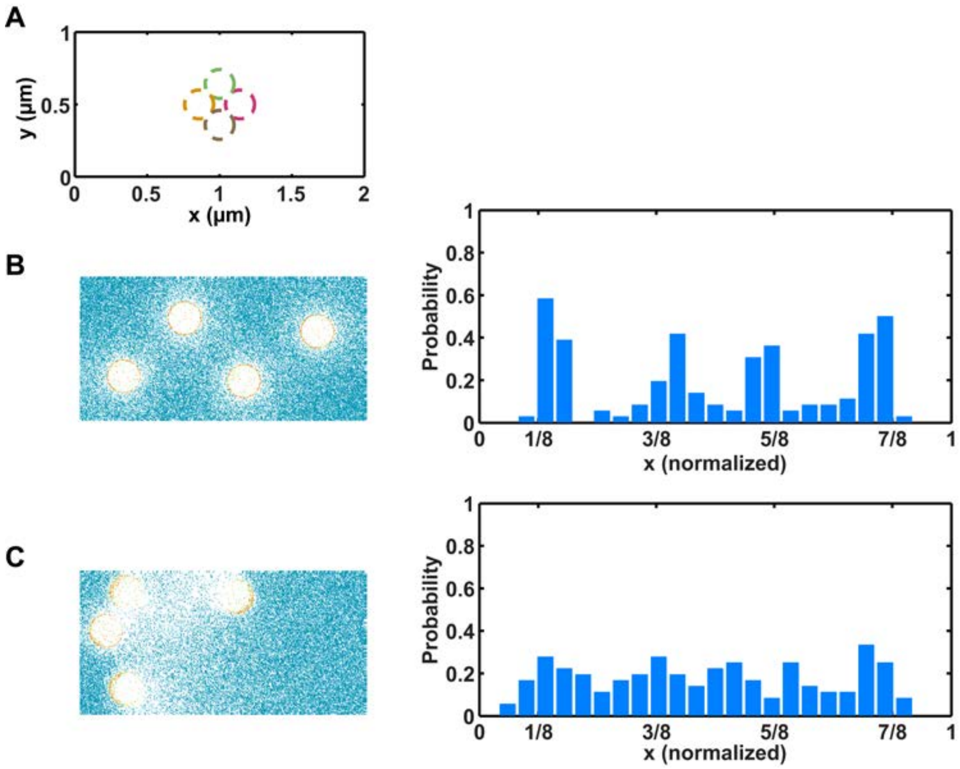

Figure S6. Impacts of PC number on PC positioning. (A) Initial configuration of 4-PC case. (B) PC distribution with a sufficient amount of ParA. (C) PC distribution with an insufficient amount of ParA. For (B-C), the ParA refilling rate, k_a, is chosen to be 0.05/sec (B) and 0.047/sec (C), respectively, with other model parameters fixed as in the Parameter table. Note that the more abundant the total amount of ParA in the system, the higher the ParA refilling rate. *Left*: snapshots of PC distribution overlapping with the ParA spatial profile. The corresponding ParA spatial profile is overlaid with the PC position in each snapshot. ParA·ATP is shown in blue-green, ParA* in green, ParA·ATP–ParB in orange, and the vacant site in white. *Right*: statistical analysis of steady-state PC distribution along the long-axis of the nucleoid. For each case, the number of simulation runs is 36.

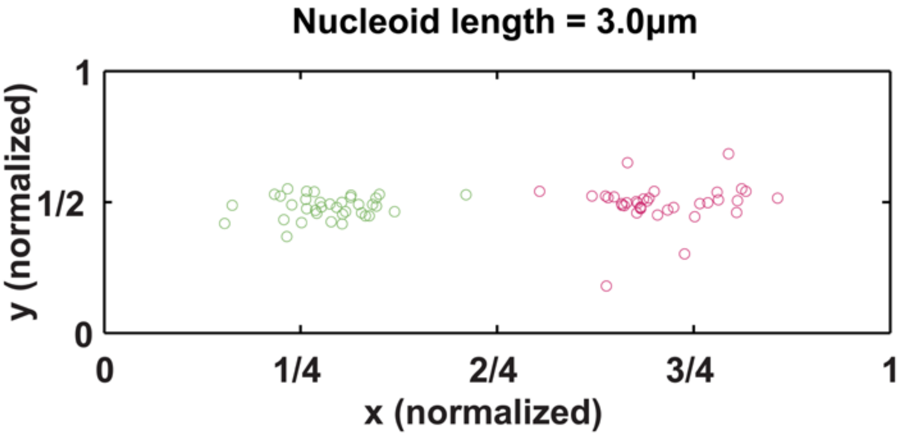

Figure S7. Partition uncertainty increases with nucleoid length. We simulate the partitioning of two partition complexes (denoted in green and magenta colors, respectively), which start from the 1/2-position on the nucleoid. We choose the bond dissociation rate as 1.2/sec and ParA refilling rate as 0.017/sec while keeping all the other model parameters fixed at their nominal values (see parameter table). The scatter plot represents the results from 36 stochastic simulation runs; it shows the steady-state positions of the two PCs after 10 mins of segregation. Importantly, when the nucleoid becomes longer, the precision in pinpointing the two partition complexes to quarter positions becomes lower (compared this result with Figure 5E), the essence of which is insensitive to the parameter choices.
